# Supplementary figures and images for: Loss of endocytosis-associated RabGEF1 causes aberrant morphogenesis and altered autophagy in photoreceptors leading to retinal degeneration
Source: PLoS Genet. 2020 Dec 23;16(12):e1009259. doi: 10.1371/journal.pgen.1009259 (PMC7790415; doi:10.1371/journal.pgen.1009259)

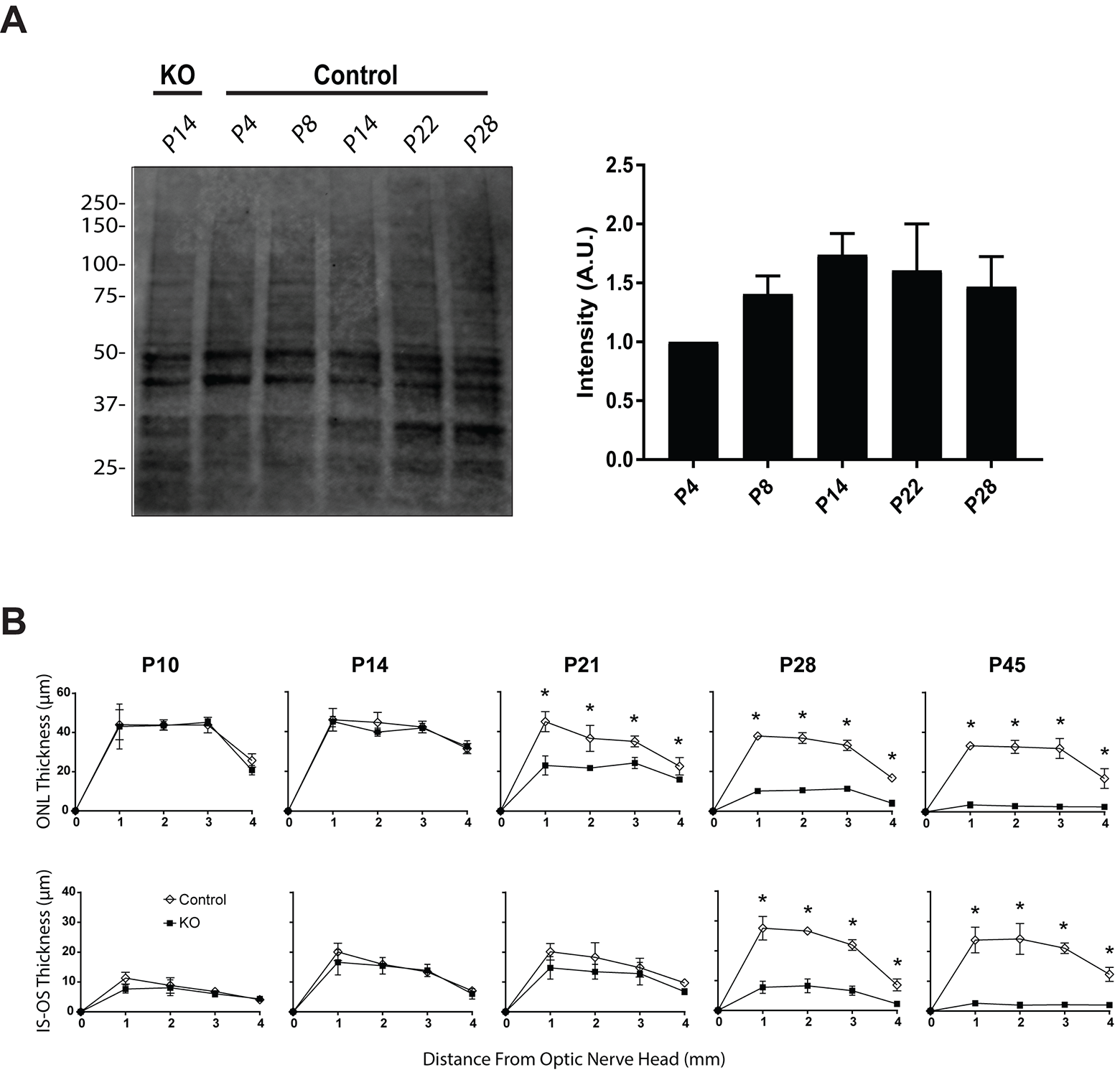

Supplement: S1 Fig — (A) Total protein loading control (left panel) for RabGEF1 immunoblot shown in Fig 1D, and quantification of total RabGEF1 expression (right panel). (B) Quantification of outer nuclear layer (ONL) thickness and inner and outer segment (IS-OS) length from hematoxylin and eosin stained sections. (TIF) [file pgen.1009259.s001.tif]

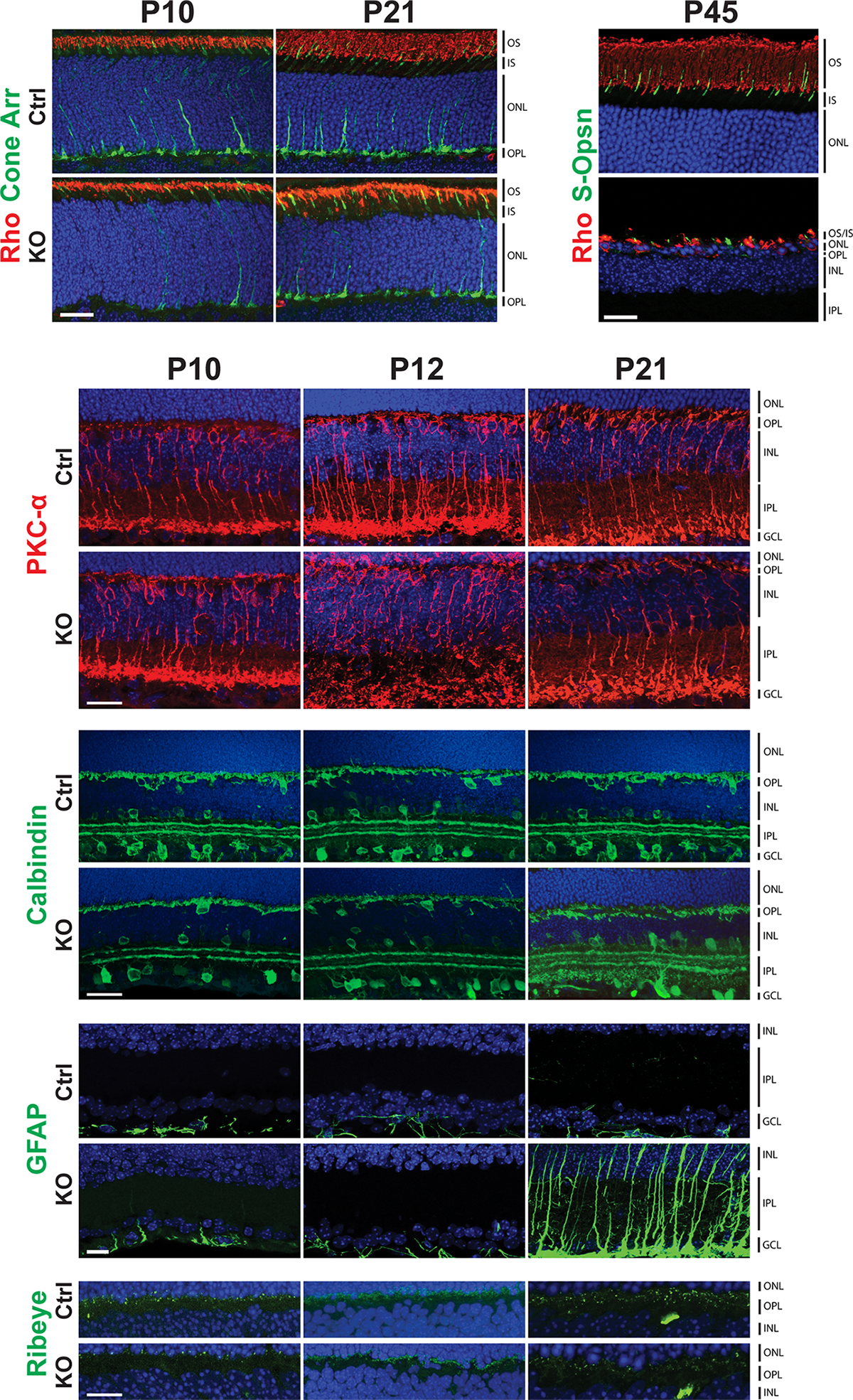

Supplement: S2 Fig — Rho (Rhodopsin, a marker of rods), Cone Arr (Cone arrestin, a marker of cones), PKC-α (a marker of ON-bipolar cells), Calbindin (a marker of Horizontal, Amacrine, and some Ganglion cells), GFAP (glial fibrillary acidic protein, marker of Müller glia stress), and Ribeye (a marker of photoreceptor ribbon synapses). Retinal sections were counterstained with DAPI to visualize nuclei. OS, outer segment; IS, inner segment; ONL, outer nuclear layer; OPL, outer plexiform layer; INL, inner nuclear layer; IPL, inner plexiform layer; GCL, ganglion cell layer. Scale bar = 20 μm. (TIF) [file pgen.1009259.s002.tif]

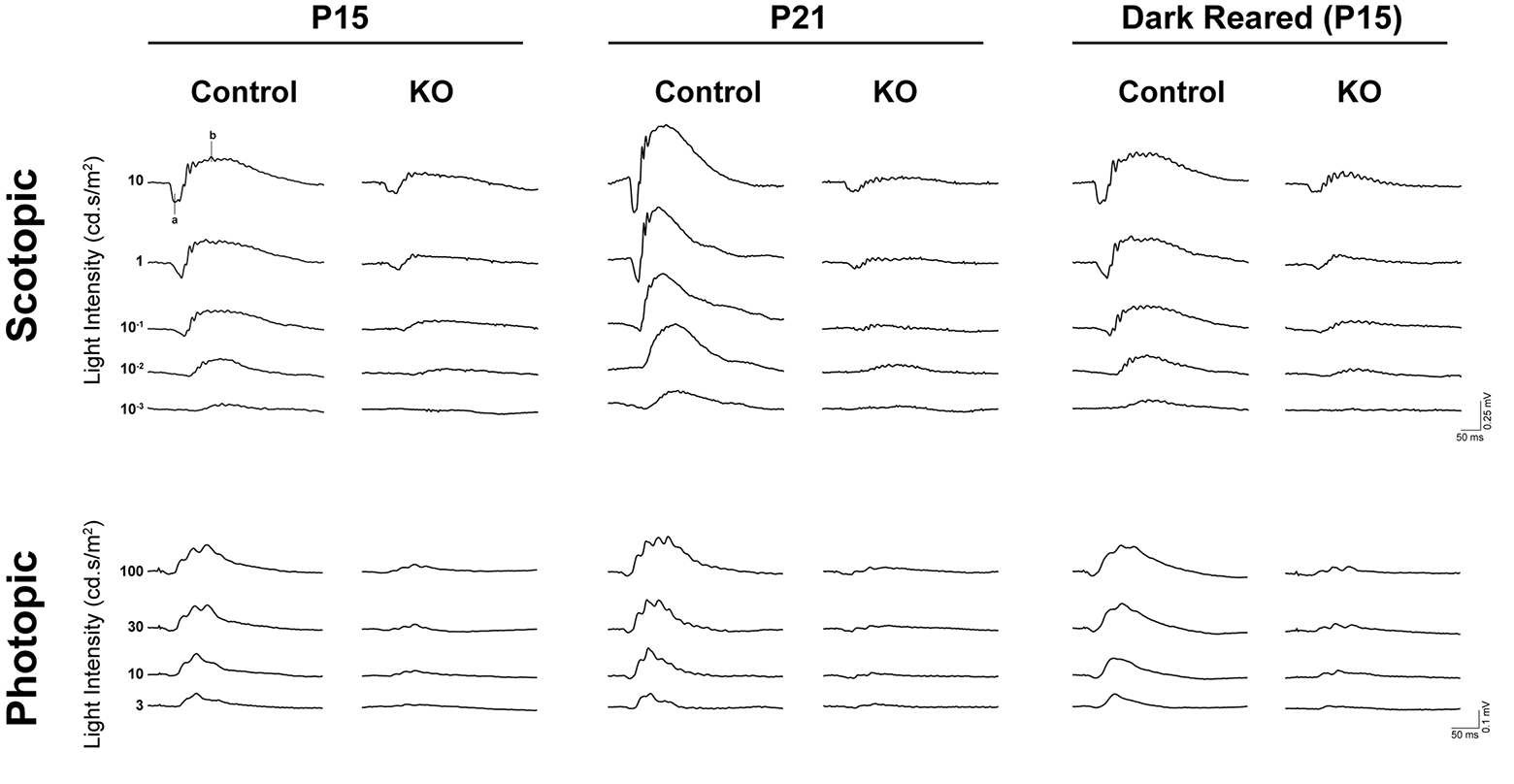

Supplement: S3 Fig — Representative traces are shown at increasing light intensities for pairs of control and Rabgef1-KO animals. Reduced function is indicated by smaller a-wave (first negative peak) and b-wave (positive peak). The a- and b-wave peaks are labelled in the top left trace for reference. (TIF) [file pgen.1009259.s003.tif]

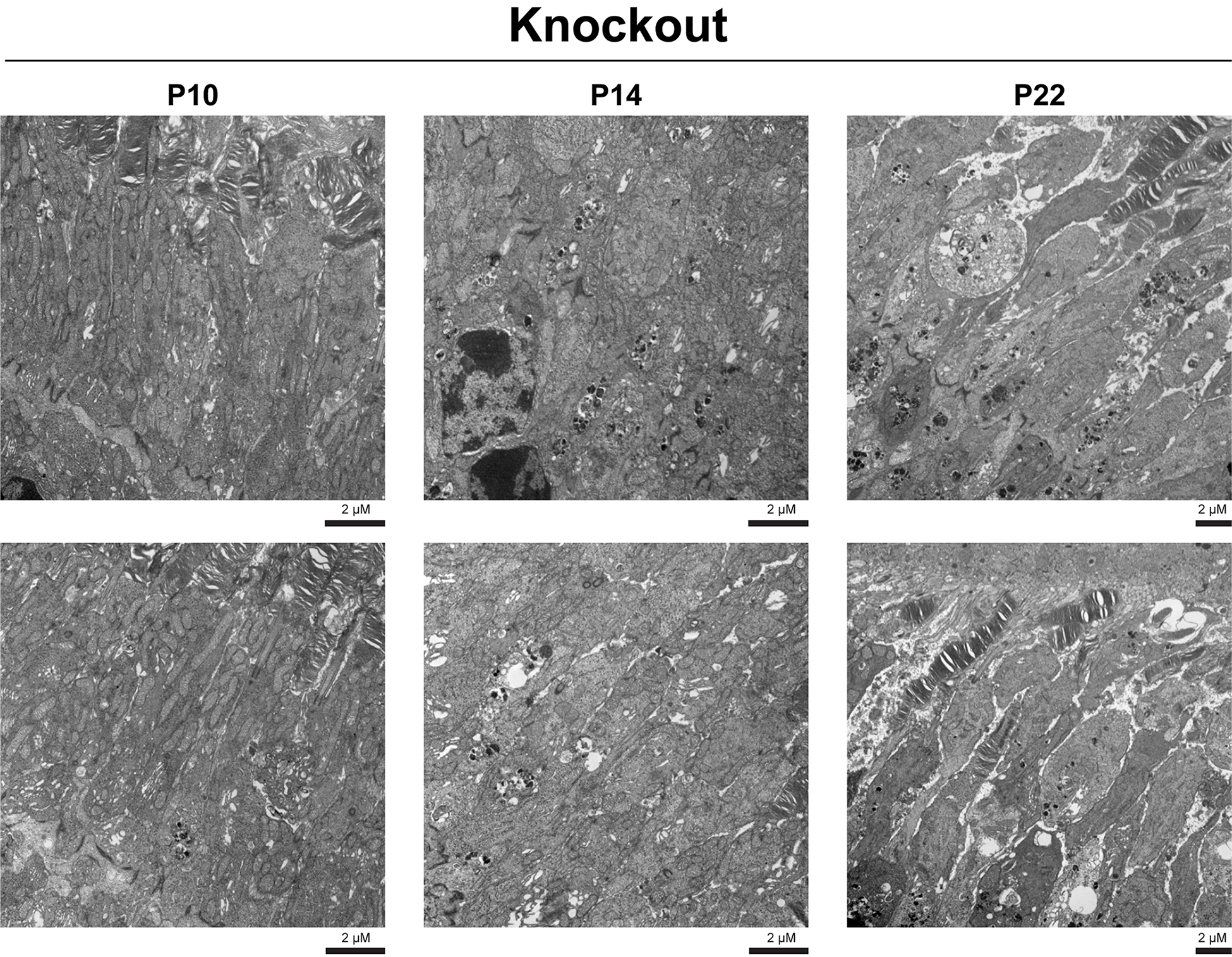

Supplement: S4 Fig — Scale bar = 2 μm. (TIF) [file pgen.1009259.s004.tif]

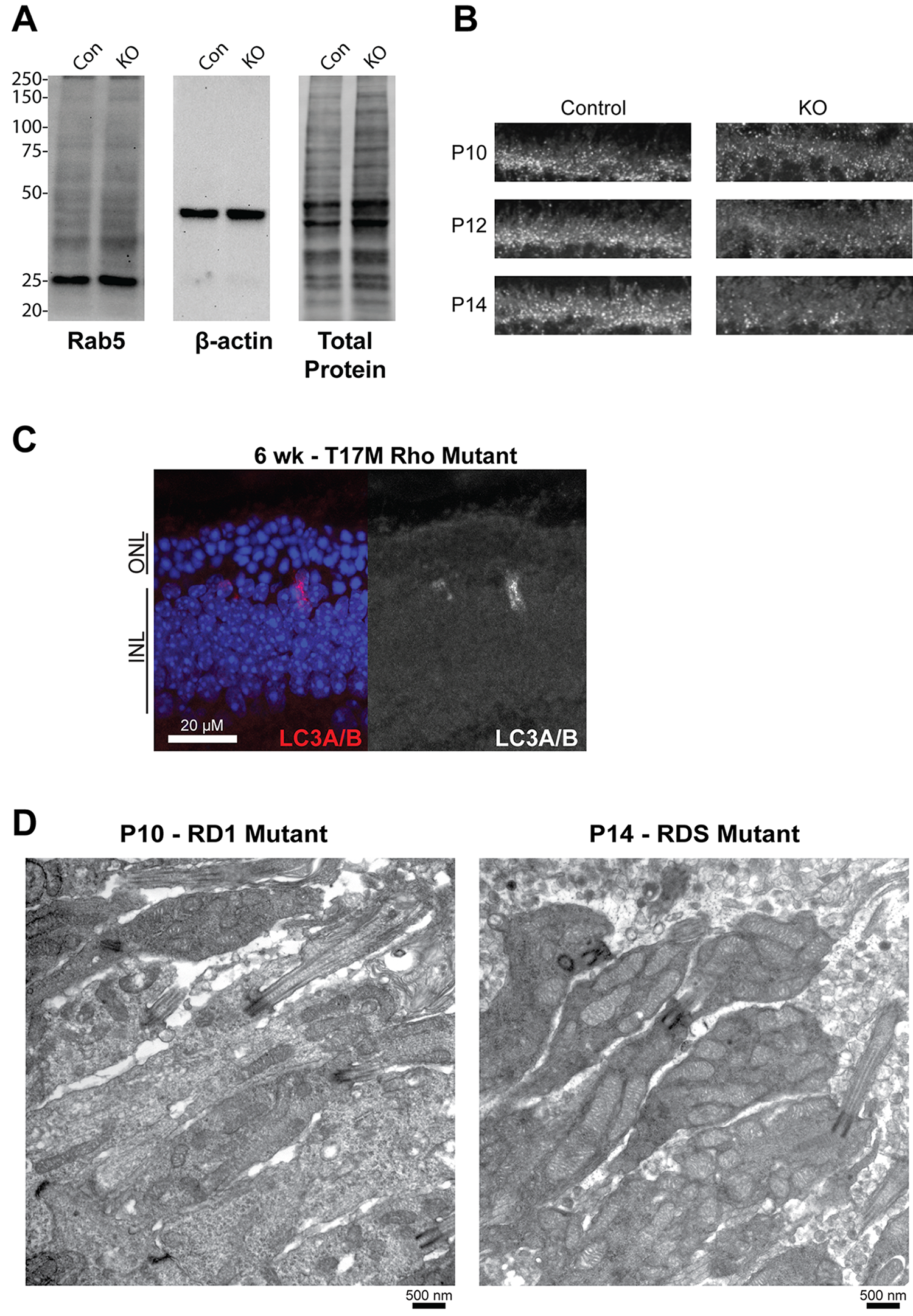

Supplement: S5 Fig — (A) Expression of Rab5 protein in Control (Con) and Rabgef1-KO (KO) animals at P14. (B) Representative EEA1 immunohistochemistry images used for quantification shown in Fig 5C and 5D. (C) Immunohistochemistry of anti-LC3A/B on Rhodopsin T17M mutant 6-week-old littermates. There is no visible accumulation of autophagosomes marked by LC3A/B. Retinal sections were counterstained with DAPI. ONL, outer nuclear layer; INL, inner nuclear layer. Scale bar = 20 μm. (D) TEM-acquired images of Rd1 and Rds mouse models of retinal degeneration. (TIF) [file pgen.1009259.s005.tif]

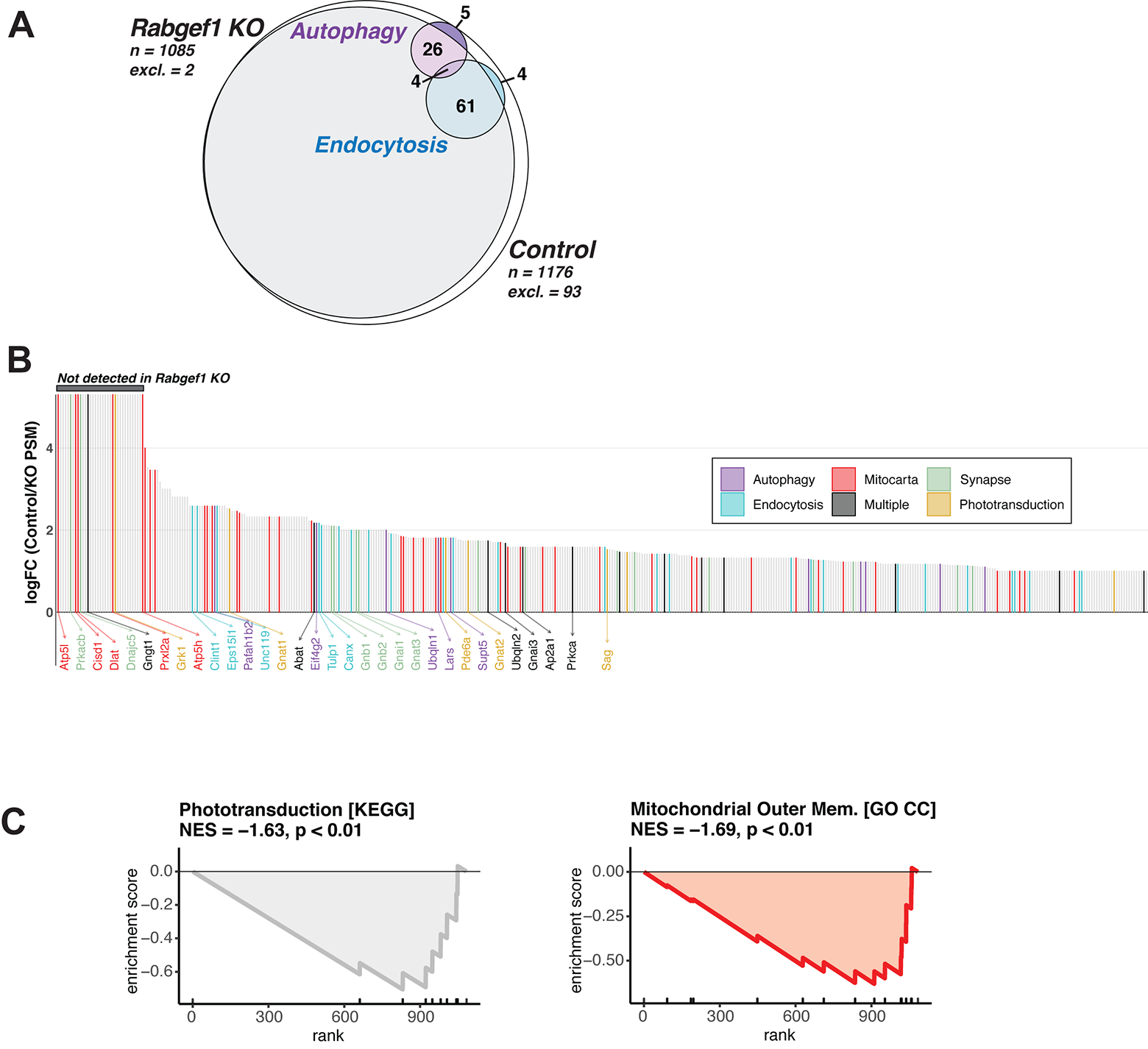

Supplement: S6 Fig — (A) Summary of proteins identified from mass spectrometric analysis of vesicle enriched sub-cellular fractions from control and Rabgef1-/- retina. The Venn diagram also shows how many of the detected proteins are annotated in endocytosis or autophagy pathways. (B) Cargo proteins enriched in control samples, ranked by decreasing order of enrichment (logFC Control/Rabgef1-/-). Proteins of Autophagy, Endocytosis, Mitocarta [64], Synapse and Phototransduction are highlighted in specific colors as described in the legend, or colored black if annotated in multiple categories. Proteins not belonging to the aforementioned groups are colored grey. (C) Enrichment plots for significant under enrichment of Phototransduction and Mitochondrial outer membrane gene sets as observed in gene set enrichment analyses of vesicular cargo from control and Rabgef1-/- retinas. (TIF) [file pgen.1009259.s006.tif]

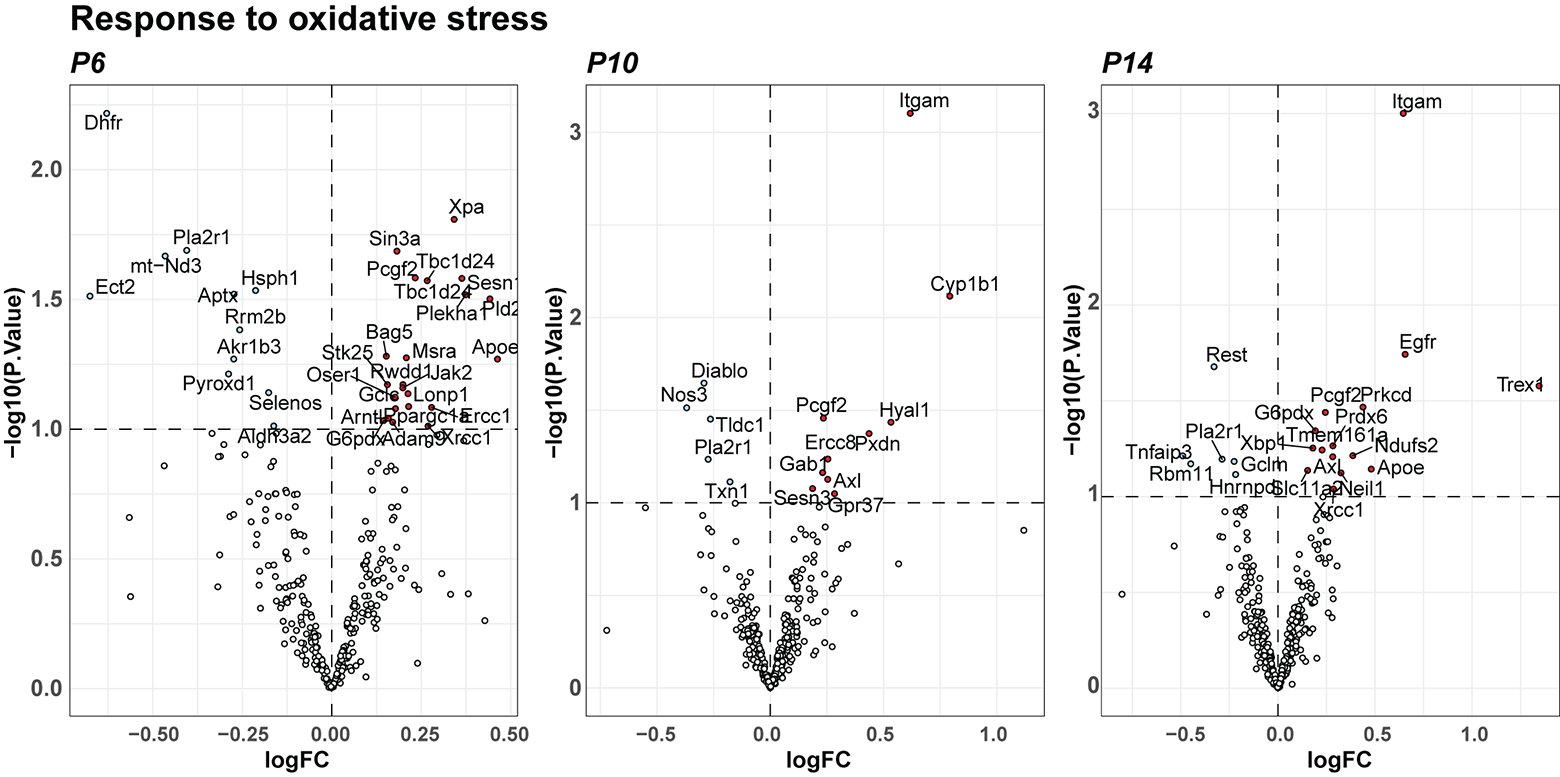

Supplement: S7 Fig — Volcano plots summarize significant differential expression of genes associated with the oxidative stress response pathway at P6, P10, and P14. Each point represents a gene where labelled or colored red and blue denote significant over-expression and under-expression in Rabgef1-KO vs WT comparisons, respectively. (TIF) [file pgen.1009259.s007.tif]

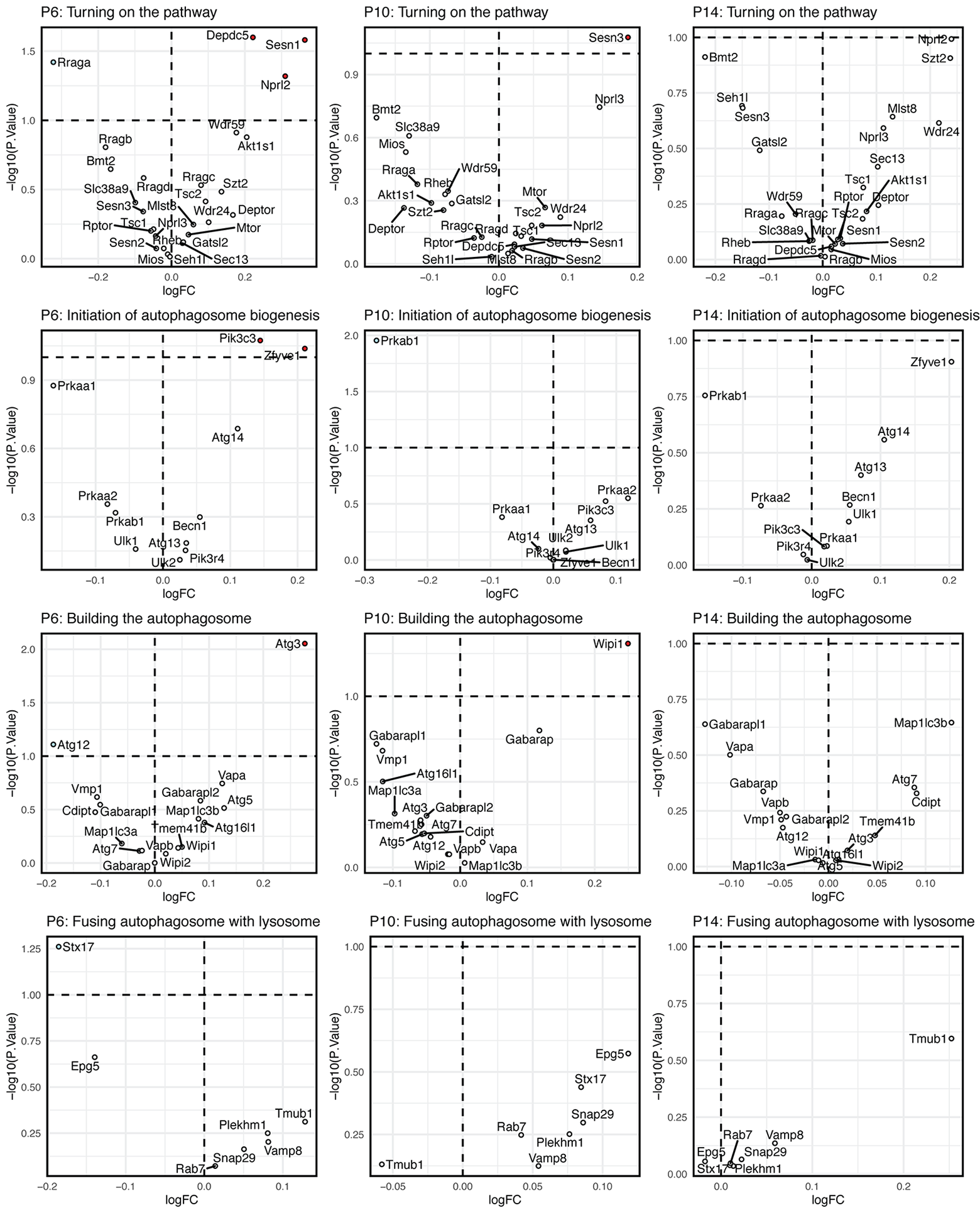

Supplement: S8 Fig — Genes participating in distinct stages of autophagy, namely: Turning on the pathway, Initiation of autophagosome biogenesis, Building the autophagosome, and Fusing autophagosome with lysosome, were obtained from [65], and investigated for their activity in the Rabgef1-KO retina. Volcano plots are arranged in a grid of time versus stage of autophagy, and a point in the volcano plots represents a single gene where colors red and blue denote significant over-expression and under-expression in Rabgef1-KO vs WT comparisons, respectively. (TIF) [file pgen.1009259.s008.tif]
