# Supplementary material for: Loss of endocytosis-associated RabGEF1 causes aberrant morphogenesis and altered autophagy in photoreceptors leading to retinal degeneration
Source: PLoS Genet. 2020 Dec 23;16(12):e1009259. doi: 10.1371/journal.pgen.1009259 (PMC7790415; doi:10.1371/journal.pgen.1009259)
Supplement: Attachment — Submitted filename: Hargrove-Grimes Response to Reviewers-09042020 Final.docx [file pgen.1009259.s012.docx]

**Response (in black text) to Reviewers’ Comments (blue)**

**Reviewer #1:** This authoritatively-written manuscript, presents evidence that depletion of Rabgef1 adversely affects photoreceptor outer segments and leads to retinal degeneration in murine mutants. Analysis of transcriptome data implicated Rabgef1 as a candidate and via experiments that extend from expression analysis, histology, electroretinography and immunohistochemistry, to TEM, IPs, MS etc., the authors demonstrate that Rabgef1 loss induces profound outer retinal phenotypes. Notably, these data are presented in a thoughtful and linear manner. The results are important and are expected to represent the foundation for future studies investigating the role of Rabgef1 in ocular disease.

We are grateful to the reviewer for generous comments.

I was unable to discern any scientific weaknesses to the data presented, and offer the following minor suggestions for textual revisions, that may enhance the publication.

1. Scope exists to enhance the background regarding the roles of Rabs, both by including a diagram/cartoon illustrating the inter-relationship of Rabs, Gefs and Gaps; and by earlier description of paralogs that induce retinal dystrophies. Although Rab27 and 28 are mentioned in the final paragraph of the discussion, more comprehensive review of this gene network would be beneficial for PLoS Genetics’s diverse readership.

We thank the reviewer for this insightful comment. We have provided an additional panel in Figure 1 to illustrate Rab GTPase cycle and specifically the relevance of Rab5/RabGEF1 in endocytic cycle. We have also added additional text about Rabs (including Rab27 and 28) in the introduction and provided a more comprehensive review of Rab5/RabGEF1 functions in the Discussion.

2. Although Rabgef1 heterozygous mice likely represent the closest model for human disease, their phenotype is unreported. Inclusion of a description would be of value, unless this would complicate a follow up study focusing on the human genetics of Rabgef1 mutation.

We did not observe a retinal degenerative phenotype in *Rabgef1* heterozygous mice (*Rabgef1^+/-^*) and have included an additional sentence in the Methods section of the manuscript to note that both heterozygous and wildtype littermates were used as controls.

RabGEF1 mutations have not been implicated in human disease as yet. However, loss of *Rabgef1* in mice has previously been shown to cause severe skin inflammation and epidermal hyperplasia. Given its key role in endocytosis, we suggest that RabGEF1 mutations will exhibit a syndromic phenotype including retinopathy. Further investigations with appropriate patient cohorts are necessary to evaluate this hypothesis.

---------------------------------------------------------------------------------------------------------

**Reviewer #2:** Using transcriptomics data, the authors have identified Rabgef1 as the only rab-associated protein that is upregulated during photoreceptor development. Given the importance of Rab function in photoreceptor function, they studied a rabgef1 knockout mouse. They find that rabgef1 knockout mice undergo retinal degeneration, although the mechanism of this retinal degeneration remains unclear. Since rabgef1 is a GEF for rab5, they suggest a mechanism related to a reduction in rab5 activity, which is supported by the reduction in EEA1 punctae that they claim to observe, however they don’t actually present any data on rab5 in their study.

We sincerely appreciate the critique of the reviewer. We have now included new immunoblotting data in Supplemental Figure 5A that shows no change in Rab5 expression in the *Rabgef1*-KO retina. We have also clarified the text to explain the rationale for probing the Rab5 activity instead.

We were not able to determine whether the absence of RabGEF1 affected the localization of Rab5 in photoreceptors since available Rab5 antibodies we tried did not work for retinal immunohistochemistry. We tested multiple antibodies, including those demonstrated to work in cell cultures and some tissues, but no specific Rab5 staining was detected in the retina sections.

Furthermore, the link between the apparent reduction in EEA1 punctae and the interesting observation of LC3-labeled punctae and autophagosomes is unclear.

We thank the reviewer for pointing us to the right direction. We apologize for the oversight and had not clarified the relationship between endocytosis and autophagy. Our further review of the literature and additional studies made us realize the direct role of RabGEF1 in autophagosome biogenesis in addition to early endosome biogenesis and fusion during endocytosis.

To further validate LC3 data, we performed new light and EM immunolabeling data with p62 (see new Fig. 6). Our studies confirmed the accumulation of both LC3 and p62 in autophagosome vacuoles suggesting a decreased autophagic flux.

We have completely revised the Discussion to clarify and explain the data and propose a model for photoreceptor cell death in Rabgef1-KO retina. We also included a previous report of early onset retinal degeneration in *Cnl6*-mutant mouse because of the disruption in autophagy-lysosomal pathway and mitochondrial turnover in rod photoreceptors.

The transcriptomic and proteomic data presented in figures 6 and 7 do not help to clarify the story. Overall, the rabgef1 knockout phenotype is interesting and the observation of increase LCA3-labeled punctae is also very interesting, but the story seems to stop at a descriptive and correlative level, and thus feels incomplete.

A general weakness is the use of whole retinal lysate for various experiments to test the specific role of RABGEF1 in photoreceptor development, despite its wide expression pattern in many retinal cell types. The RNA-seq data also stem from total retinal RNA, thus hindering specific relevance to photoreceptors.

We agree that a better experiment would be to perform RNA sequencing on individual flow-sorted photoreceptors. We attempted this experiment by crossing our Rabgef1 strain with NRL-GFP mouse that expresses GFP in all rod photoreceptors that can then be used for flow sorting. However, the reporter strain is on a C57BL/6 background and we were unable to obtain live Rabgef1-KO births. Dr. Galli’s lab, from whom we obtained the Rabgef1 heterozygous mice, have confirmed that they were unable to obtain live KO births on a largely C57BL/6 background either. Currently, the Rabgef1 line is mostly BALB/C, which has allowed us to obtain complete null mice, and even then, we obtain fewer than the 25% expected from a heterozygous x heterozygous cross. We would like to point out that rod photoreceptors constitute around 75% of all cells in the mouse retina, and therefore our RNA sequencing data largely represents gene profiles of rod photoreceptors. Figure 1B and C indicate that Rabgef1 has an expression pattern that matches that of rod differentiation. We have also included lower magnification confocal images in the revised Figure 6 showing that autophagy defects we observed are restricted to photoreceptors. No defect was evident in other retinal cell types.

We appreciate the reviewer’s critique and apologize for the lack of clarity in explaining our transcriptomic and proteomic studies and its possible link to the phenotype.

Please see pages 10-11 and 13-14 for revised Results and Discussion.

Specific points:
Page 7, paragraph 2-It would be useful to have some quantification of the photoreceptor phenotype, possibly an actual measurement of outer segment length.

We have provided additional data in Supplemental Figure 1B where we have quantified both the outer nuclear layer and the inner/outer segment thickness of P10 – P45 mice.

Page 8, paragraph 2 (Figure 5)-The authors claim there was much less EEA1 immunoreactivity in the KO than in the control. It would be useful for the authors to mention how this quantification was done. If it was performed on a single Z-slice, as is suggested, was the same Z-slice used for both conditions, and how was it chosen? Is it possible that the knockout has EEA1 in a different retinal slice than the wild type? Also it would be useful for the authors to show the full western blot from Figure 5E.

Thank you allowing us to clarify how the quantitation was performed. The EEA1 puncta were quantified from multiple single slices collected as Z-stacks, and the number of slices and imaging intervals were identical in both control and KO sections. A description of the assay that includes these details has been included in the manuscript. We have also replaced the cropped EEA1 immunoblot in Figure 5E with a full blot.

Figure 6-The increase in LC3A/B-labeled punctae in the knockout is striking. Perhaps the authors **could show the full western blot from Figure 6C**, to confirm the specificity of their antibody?

We have provided an additional full western blot of LC3A/B in Figure 6C.

Page 9, paragraph 2-the authors looked at endosomal vesicular fractions, which they argued should be enriched in autophagosomes, and observed a decrease in phototransduction proteins and mitochondrial homeostasis proteins in their Rabgef1 knockouts relative to the controls. If there were an increase in autophagosomes, wouldn’t you expect the opposite? But then again, does this quantification really mean anything, given the decrease in number of photoreceptors in the knockout? At what age was the quantification performed?

We pooled whole retina samples (n=20 per genotype) from ages P14-P19, ages where LC3A puncta are observed but before substantial degeneration and cell loss occur.

We apologize. The reviewer is indeed correct. The increase in LC3A/B and p62 (new data) is most consistent with decreased autophagic flux and reduced degradation of macromolecules. We have completely rewritten the Discussion to clarify these points.

Overall, the proteomic and transcriptomic analysis in Figure 6 and 7 don’t seem to add much to the paper, and don’t really help to explain the phenotype.

We agree with the reviewer’s criticism and apologize for the lack of clarity and oversight in explaining the proteomic and transcriptomic studies.

From immunohistochemical studies, we observed an increase in LC3 (autophagosome membrane component) as well as p62 (adaptor of specific cargoes for autophagy) in Rabgef1 mutants. Together they indicate accumulation of autophagosome vacuoles with of amorphous cargo of macromolecule aggregates due to blockage in their degradation. The proteomic data revealed the presence of specific proteins that were accumulated in autophagosome-enriched vesicle fraction and were not recycled or degraded. A decrease in phototransduction, mitochondrial and synaptic proteins in the KO retina fraction likely due to reduced expression and/or less recycling of these proteins. Given that this analysis does not provide significant insights, we have now transferred this to the Supplement.

Transcriptome analysis of KO retina at developmental stages before the accumulation of autophagosomes and photoreceptor cell death, however, unraveled new insights into the possible trajectory of cellular events leading to compromised autophagy. We have now revised the Results and Discussion sections thoroughly to clarify these studies.

Rather than including all the changes here, we would request the reviewer to please see pages 10-11 and 13-14.

The most striking result in the study is the increase in LC3A/B labeled punctae in Figure 6. It would be interesting to know, however, whether the objects, claimed to be autophagic structures (Fig. 4), can be labeled with an autophagic marker, such as LC3.

We have now included p62 Airyscan and immuno-EM data, which closely resembles the electron-dense structures observed in the inner segments of KO photoreceptors. Please see the new Fig. 6 and pages 9-10. Discussion now explains the data more clearly and provides a model for cell death.

……

**Reviewer #3:** On early endosomes, activation of Rab5 is catalyzed by the GEF Rabex5 complexed with the effector Rabaptin5 contributing to early endosome generation and trafficking.In the current paper, with the title ‚Loss of endocytosis-associated guanine nucleotide exchange factor RABGEF1 causes defects in photoreceptor outer segment biogenesis leading to retinal degeneration‘, Hargrove-Grimes et al show, that loss of Rabgef1/Rabex5 in mice resulted in a failure of sufficient early endosome production and endosome trafficking leading to an upregulation of autophagy in the retina and defects of retinal photoreceptor morphology. Using the RABGEF1 KO mouse, the authors show that RABGEF1 is necessary for rod photoreceptor function and maintenance. As cause for progressive degeneration starting after P14, the authors discuss defects in endosome formation leading to an increase in autophagosome formation suggesting autophagy malfunction.The study suggests an essential role of the endocytic machinery in photoreceptor outer segment biogenesis and function as absence of Rabex5 resulted in an almost complete loss of both rod and cone function early on.
Thes study is shedding light on the importance of the endocytic machinery in delivering cargo for ciliary trafficking of photoreceptors towards outer segment generation. As such I regard it as principally well suited to be published in PLoS Genetics.

We thank the reviewer for the kind comments.

There are some criticisms which should be addressed before publication:

A general shortcoming of the study is its focus on rod photoreceptors, disregarding the cones. The manuscript would greatly increase significance, if the cone phenotype would be analyzed after loss of Rabgef1/Rabex5 in mice. Please include data cone opsin trafficking and cone behavior. Is for example cone death a primary event or a consequence of rod death (secondary cone degeneration). Figure 3 shows the reduction of rhodopsin but there is no data on cone opsins. What is the situation there, given that both a and b wave are greatly reduced?

We thank the reviewer for noting this oversight. We have included cone arrestin data to Supplemental Fig. 2. Additional immunohistochemistry data indicate that loss of *Rabgef1* does not result in a detectable abnormalities in other non-photoreceptor cell types (bipolar cells, amacrine cells, horizontal cells and RGCs).

Both rod and cone photoreceptors are impacted; however, cones in general tend to survive much longer in most models of retinal degeneration. A previous report has also shown that disruption of autophagy-lysosomal pathway in rods can lead to photoreceptor cell death in *Cln6*-mutant mice. Please see Discussion pages 12-14.

Figure 1B: A negative control either using a sense probe or the P21 KO would be helpful to distinguish between background and specific staining.

We have included an additional panel in Figure 1 showing the absence of staining using the same probe in KO tissue.

Figure 3B and corresponding text: The blots suggest a delay in protein expression, which does not fit to the discussed ‘decreased recycling of membrane proteins’. In the transcriptome dataset a downregulation of phototransduction genes. How do the authors explain/discuss this early effect of RABGEF1-KO, which happens before photoreceptor-specific RABGEF1 expression is upregulated?

We have modified the text and removed ‘delay in protein expression’. We do not believe there is a delay in protein expression, as the Rabgef1 KO animals’ protein expression never quite ‘catches up’ to control levels. There is much less protein at all stages of development in the Rabgef1 KO retina compared to controls. We have also replaced ‘decreased recycling of membrane proteins’ with decreased levels of membrane proteins since we don’t have any direct evidence to show that recycling of these proteins was altered.

Our published time series transcriptomic data from developing whole retinas as well as flow sorted rods (Suppl Table S1) demonstrate that Rabgef1 expression is substantial (30-40 CPM) at and before P6. This indicates its function even before the dramatic upregulation of morphogenesis and function-associated genes in photoreceptors. In this study, we performed RNAseq experiments to obtain insights into the early changes caused by loss of Rabgef1 before the degeneration begins. Our analysis revealed misregulation of cell cycle and phototransduction genes at P6 indicative of differentiation defects and stunted growth of cilia (outer segment) implying aberrant rod morphogenesis. These observations correlate with our TEM and histological studies and indicate that absence of Rabgef1 leads to detrimental changes long before cell death is evident.

Figure 5C: In P10 samples, more EEA1 staining seems to be in the ONL, which was not quantified. Is there a mis-localization of EEA1-positive endosomes which results in less positive endosomes in the IS? It would be good to highlight the area taken for measurements for Figure 5D. A close-up of the selected area showing single EEA1-positive endosomes is needed to strengthen data shown in 5D. In the Figure legend title there is a typo with RABGEG1 instead of RABGEF1.

Because removal of Rabgef1 appears to only affect photoreceptors, we focused on the apical photoreceptor inner segment and quantified the number of EEA1-positive endosomes. We have added ROI boxes to Figure 5 to indicate the region that was quantified and included a more detailed description of the assay in the manuscript. We have also included higher magnification images of the ROIs shown in Figure 5 in Suppl. Fig. 5B. The typo has been corrected.

Suppl. Table S1 and S2: Please state which stages were used (P21?) in the legend.

We apologize for this oversight and the ages for RNA sequencing and Mass Spectrometry data are included in the legend.

Rabex5/RABGEF1 and other components of the endocytic pathway may however, be as essential for overall development of a vertebrate organisms, that they may not show up as potential candidates for diseases that include retinopathy phenotypes.

We agree, and we are not claiming that *Rabgef1* mutations or mutations in other endocytic pathway components will directly lead to retinal disease. As explained in response to Reviewer 1’s last comment, no autosomal dominant or recessive human disease (to our knowledge) has yet been associated with RABGEF1. This would indicate that (i) RABGEF1 is essential for normal development and/or survival, or (ii) RABGEF1 mutations cause a syndromic phenotype and one needs the right patient cohort. Based on our data, we are suggesting that the latter syndromic phenotypes would likely include retinopathy. We have revised the text and clarified it in the Discussion.

Suppl. Table S2, Figure 5B and Figure 6D-F and corresponding methods section: How many replicates did you measure? If only one, at least two more biological replicates should be prepared and statistically validated.

Thank you for noting this oversight. We have now included this information in the manuscript. The data provided in Suppl. Table S2 and revised Suppl. Figure S6 (vesicular fractions) are the result of one proteomics experiment that included 20 pooled control and 20 pooled KO retinas, respectively from mice ages P14-P19. Given that the KO retinal tissue was difficult to obtain, we were unable to repeat this experiment a second time. We have therefore transferred this data to the Supplement.

Figure 5B is the combined results of 2 independent (biological replicates) experiments. Due to the limited number of KO mice that are born and survive to P21, we were unable to perform mass spec on our co-IP samples for a third time. Instead, we performed additional immunoblots to confirm the interaction of Rabgef1 and Rabaptin5. Based on the plethora of literature in this area indicating that Rabaptin5 is an obligatory dimer with Rabgef1, we are fairly convinced that this interaction takes place in the mammalian retina as well.

Figure 6D-F: How did you perform the isolation of vesicular fractions? This part is missing in the method section.

We have included the general method used to isolate the vesicular fractions in the method section.
