# Supplementary material for: Loss of endocytosis-associated RabGEF1 causes aberrant morphogenesis and altered autophagy in photoreceptors leading to retinal degeneration
Source: PLoS Genet. 2020 Dec 23;16(12):e1009259. doi: 10.1371/journal.pgen.1009259 (PMC7790415; doi:10.1371/journal.pgen.1009259)
Supplement: Attachment — Submitted filename: Hargrove-Grimes Plos Genetics Response Revision 11072020.docx [file pgen.1009259.s013.docx]

**Response to The Editorial and Reviewer 2 Comments**

The authors have addressed the minor points from reviewer and most of the critical concerns from reviewer 2 and 3 the reviewers. Reviewer 2 accepted to review the revised version and states that most of her/his concerns can been ruled out, now and that the revised manuscript has very much improved. I agree with this and would like to extend this to the criticisms of the 3rd reviewer, who insisted on a major revision, but unfortunately did not re-examine the revised manuscript.

Thank you. We are grateful to the editor and Reviewer #2 for reading the revised manuscript.

The 3rd reviewer asked for further comments on the cone phenotype in Rabgef1-/- mice. The authors added immunostaining for cone arrestin in a supplement figure, but do not mention or comment the results in the text of the manuscript. I suggest comment the findings on cone staining in a revised version.

The original draft of our manuscript specified a defect in rod photoreceptors and did not describe cones. We have added additional text (page 7, top page 9). Immunofluorescence staining using cone marker S-opsin identified cone loss only at P45 (now included in Suppl. Fig. S2). However, TEM of P22 retina showed the presence of protein aggregates even in cone inner segments (see Fig. 4), consistent with the ERG data demonstrating cone dysfunction (See Fig. 2). Thus, both photoreceptors are affected in the RabGef1-KO mice.

We have clarified it in the text.

I also agree that the new title reflects their findings better. Nevertheless, I suggest shortening of the title.

Thank you. We have shortened the title per your request.

**Reviewer #2: Comments:**
The authors have addressed some of the critical concerns, and the paper is very much improved.
I agree with the main conclusions that 1) Rabgef1KO leads to photoreceptor defects. 2) These defects appear to be related to reduced EEA1+ early endosomes and accumulation of LC3+ autophagosomes.

Thank you very much.

I have some remaining concerns, with respect to the new Fig. 6 and Fig. 7.Fig. 6B & C are inconsistent with each other: Fig.6C, western blot, shows barely any difference in LC3A/B – I & II between control and KO animals at P12 and P14; especially if the slight increase for the KO in the tubulin loading control is accounted for. This is inconsistent with the IF result (panel B), which shows absolutely no LC3A/B signal in the control retina in any retinal cell layers, and yet there is a significant signal in the photoreceptor layer of the KO retina, thus indicating a large control v KO difference. The IF and immunoEM of p62 is striking. But, especially given the inconsistency in A and B, a western blot should be given for p62.

We thank you for allowing us to clarify Figures 6 and 7. The differences between the IF and WB results are likely due to the fact that IF shows an increase in staining for LC3 and p62 only in a subset of photoreceptor cells (see Fig. 6A,B,D), whereas WB measures levels of these proteins in whole retinal extracts (Fig. 6C, F). In addition to photoreceptors, the retina contains other cell types in which Rabgef1 KO does not cause accumulation of LC3 and p62, as observed by IF. Thus, the signal of increased LC3 and p62 in the subset of photoreceptor cells is diluted in whole retinal lysates, to the point that differences are not detectable by WB analysis. This interpretation is now explained in the text.

The transcriptome analysis figure (Fig.7) does not strongly support the autophagy-related conclusions and perhaps should not be left as a main figure.Fig. 7B: It appears that the strongest changes are at P6, and in functions related to cell-cycle regulation.Fig. 7C,D,E: Many of the highlighted genes, while suggesting significant differential expression (pValue < 0.05, -Log10 >1.3), are, nevertheless, not changed much, as their LogFC values are mostly below 0.75 (Log2=0.75), which means there is less than a 2.

We welcome the reviewer’s comment but feel that the transcriptome analysis is an important part of this study. The transcriptomic evaluation was intended to elucidate the events leading to photoreceptor cell death in the absence of Rabgef1. It is important to note that the retinal photoreceptors are still viable and apparently normal at the early sampling timepoints, and therefore we anticipated to see only moderate yet statistically significant differential expression. While the P6 shift of the cell cycle pathways was striking, the concurrent abnormality in phototransduction and Oxphos at P6 and P10 illustrate the failure of normal photoreceptor development, which we predict adds to cellular distress. Another remarkable observation comes at P14 when compensatory mechanisms become prominent as the KO-retina upregulates lysosome and early endosomal genes. These trends highlight the broader importance of Rabgef1 in retinal development and sustainability. Our transcriptome analysis thus allows to see the very early events and compare with the well characterized photoreceptor development program which has a strong gene expression basis. We therefore would like to retain Fig 7 as a main figure.

Supplemental Fig. S5B is not mentioned anywhere in the manuscript.

Thanks. Figure S5B is mentioned in the revised manuscript on **page 10.**

Supplemental Fig. S5C two-colored image on the left does not seem to match the single-colored image on the right – there is clear red signal in at least 2-3 places in the image on the left, yet none is seen in the right image.

This was a single image with the left half shown in grayscale. We agree that our previous panel was confusing, and this has been corrected. The revision now shows the same region of the retina in both panels, with the left panel shown in color with DAPI and right panel depicting the grayscale image of the red channel.

Supplemental Fig. S8: if I understood it correctly, the y-axis is showing the –log10 calculation of respective pValue for each of the differentially expressed genes, between Ctrl and KO retinas, highlighted in the graph. If that is the case, most of the genes listed here were not actually differentially expressed between the two genotypes: pValue of 0.05 would yield a –log10 value of around 1.3. Therefore, anything below 1.3 would not be considered significantly differentially expressed. Only 4 or 5 genes in the autophagosome-related pathways were differentially expressed at P6 and perhaps only 1 at P10 and none at P14. I don’t think the gene expression data is very convincing here. The perturbation of autophagy in the KO animal could be real, but it is possible that this is more reflected at the protein level rather than the transcriptome level.

Yes, the reviewer’s understanding of the figures is correct and indeed autophagosome genes show little differential gene expression in the times preceding photoreceptor cell death. We agree with the reviewer in thinking that the regulation of autophagy may be happening at protein level or via signaling events such as phosphorylation. The corresponding result has now been modified to reflect this interpretation.
